# Targeting PI3K/AKT/mTOR Signaling Pathway as a Radiosensitization in Head and Neck Squamous Cell Carcinomas

**DOI:** 10.3390/ijms232415749

**Published:** 2022-12-12

**Authors:** Yu-Chieh Su, Wei-Chang Lee, Chih-Chun Wang, Shyh-An Yeh, Wen-Hui Chen, Po-Jen Chen

**Affiliations:** 1 Department of Medicine, School of Medicine, I-Shou University, Kaohsiung 840203, Taiwan; 2 Division of Hematology-Oncology, Department of Internal Medicine, E-Da Hospital, Kaohsiung 824410, Taiwan; 3 Department of Otolaryngology, E-Da Hospital, Kaohsiung 824410, Taiwan; 4 Department of Radiation Oncology, E-Da Hospital, Kaohsiung 824410, Taiwan; 5 Department of Medical Imaging and Radiological Sciences, I-Shou University, Kaohsiung 840203, Taiwan; 6 Department of Dentistry, E-Da Hospital, Kaohsiung 824410, Taiwan; 7 Department of Medical Research, E-Da Hospital, Kaohsiung 824410, Taiwan

**Keywords:** head and neck cancer, radiotherapy, PI3K/AKT/mTOR, radiosensitization

## Abstract

Globally, there are over half a million new patients with head and neck squamous cell carcinomas (HNSCC) every year. The current therapeutic approaches to HNSCC are surgery and adjuvant radiotherapy. These approaches carry a high incidence of metastasis or recurrence from HNSCC cells’ radioresistance. Recent studies have revealed that a combination with radiosensitizers can be used to improve the radioresistance in HNSCC; however, few agents are approved as radiosensitizers. The constitutive activation of phosphatidylinositol-3-kinase (PI3K)/protein kinase B (AKT)/mammalian target of rapamycin (mTOR) pathway is a vitally oncogenic type of signaling that promotes tumorigenesis, metastasis, and radiotherapy resistance in HNSCC. Pharmacological targeting of PI3K/AKT/mTOR signaling pathway is considered a promising strategy of radiosensitization in HNSCC. In this review, we summarize the oncogenic significance of PI3K/AKT/mTOR signaling in HNSCC with radiotherapy resistance and highlight the therapeutic potential of small molecule inhibitors against PI3K/AKT/mTOR signaling for the radiosensitization in HNSCC treatment. It provides a mechanistic framework for the development of new drugs for radiosensitization in HNSCC radiotherapy via targeting PI3K/AKT/mTOR signaling pathway.

## 1. Radiation Therapy in Head and Neck Cancer

Cancer has been the leading cause of death for many years. Head and neck squamous cell carcinoma (HNSCC) is the fifth most common cancer in men and the fifth leading cause of cancer death in men, with 650,000 new cases and 350,000 deaths annually globally [1,2,3,4,5]. Smoking; alcohol consumption; infection with human papilloma, particularly serotype 16; and betel quid chewing are all known risk factors for HNSCC. Improvements in the long-term survival rate of patients with HNSCC have stagnated in recent years. It has been proposed that reliable prognostic factors could assist in the identification of high-risk groups by upgrading the TNM staging system [6].

HNSCC is treated locally through surgical resection. If surgery is impossible, one of the most prominent local cancer treatment methods is radiation therapy. Radiation therapy is used as an adjunct or palliative treatment for many cancers. Although RT is an effective treatment for oral cancer, tumor cell resistance to this treatment is a major concern. Radiosensitizers are drugs that, when integrated with radiotherapy, increase the cell-killing effect of radiotherapy. However, only a few chemotherapy drugs (such as cisplatin and carboplatin) have had this effect so far, and even in recent years, radiosensitizers have made slow progress [7].

The treatment for HNSCC is usually radiotherapy and/or surgery. The selection and success of these treatments depend on the resources and knowledge held by the medical service provider. Surgery removes cancer tissue as well surrounding lymph nodes. This is usually followed by adjuvant or neoadjuvant radiotherapy, chemotherapy, or combined radio chemotherapy [8,9,10,11,12]. These follow-up treatments are associated with several severe side effects, including mucositis, dysphagia, leukopenia, and thrombocytopenia. These side effects not only increase the likelihood of infection and bleeding but also reduce the quality of life experienced by the patient [9,13,14,15,16]. 

Previous research showed that induction chemotherapy with cisplatin and 5-FU before radiotherapy or radio-chemotherapy improves overall survival in patients with advanced HNSCCs, which not only increases antitumor activity but also reduces the concentration of treatment administration [9,17,18], although the induction chemotherapy may compromise further locoregional approaches with an increase in side effects and decrease in tolerance [19]. Therefore, it is desirable to develop radiosensitizers for the treatment of patients with radioresistant HNSCC.

## 2. Significance of PI3K/AKT/mTOR Signaling on Radioresistance

It has been found that the phosphatidylinositol-3-kinase (PI3K)/protein kinase B (AKT)/mammalian target of rapamycin (mTOR) signaling pathway is overstimulated in cancer cells that are resistant to chemotherapy, radiation, and hormone therapy [20]. There is some evidence that irradiation resistance in various cancer cell types can be reduced in vitro and in in vivo xenograft models of cancer by dual targeting of PI3K and mTOR [21,22,23]. In both recurrence and non-recurrence groups from patients with HNSCC who had previously received definitive surgery and adjuvant radiotherapy, high phosphorylation levels of PI3K, AKT, mTOR, eukaryotic initiation factor 4E (eIF4E), and eIF4E binding protein 1 (4EBP1) were observed [24,25]. Studies in HNSCC patients have revealed a similar phenomenon, indicating that overexpressed proteins in the PI3K/AKT/mTOR signaling pathway, such as p-mTOR and p-AKT, may be used as good prognostic biomarkers [26]. 

**Figure 1:**
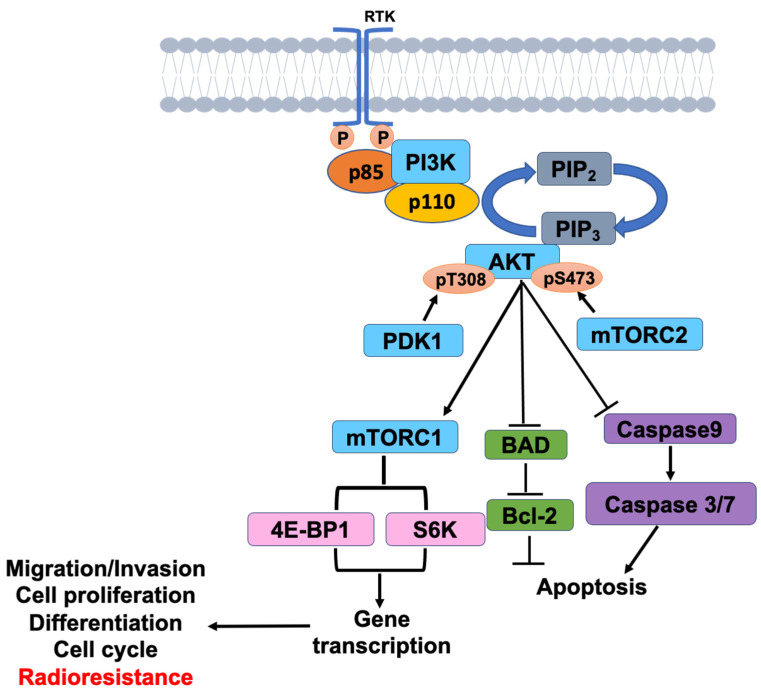
PI3K/AKT/mTOR signaling in HNSCC.

The PI3K/AKT/mTOR signaling pathway has been linked to radiotherapy resistance, and the effect of inhibitors of this pathway as cancer radiosensitization is worth investigating [27,28]. The activation of PI3K/AKT/mTOR pathway is the primary mechanism causing cancer cells to develop radiation resistance. When PI3K is activated, it produces PI(3,4,5)P3 at the D3 position of the PI(4,5)P2 inositol ring of the plasma membrane, PIP3, and the signal protein AKT/PDK1 containing pleckstrin homology (PH domain) in the cell (phosphoinositide-dependent kinase-1) binding, leading to AKT activation. Through phosphorylation, activated AKT activates downstream mTOR or inhibits Bad, Caspase 9, and other proteins, regulating cell proliferation, differentiation, apoptosis, and migration (Figure 1). The PI3K/AKT/mTOR signaling pathway has been thoroughly researched and has been shown to be important for RT resistance in different cancer types [29,30,31]. Previous research has shown radiosensitization in response to dual PI3K/mTOR inhibitor treatment (in other cancer cell lines, both in vitro and in vivo) [32,33], but an in vitro condition does not adequately depict clinical scenarios. 

Although radiation therapy is an effective treatment for oral cancer, tumor cell resistance to this treatment remains a major concern. Understanding the molecular mechanisms involved in tumor sensitivity to irradiation, which leads to radioresistance, and identifying targets for its therapeutic use are therefore critical for improving radiation therapy and overcoming radioresistance [29,30,31]. Merging dual PI3K/mTOR inhibitors and irradiation treatment has a synergistic anticancer effect on OSCC (oral squamous cell carcinoma); however, this has only been partially investigated and is mostly unknown.

The PI3K/AKT/mTOR axis is essential for regulating cell growth migration, survival, and protein synthesis. Particularly, mRNA translation and cell-cycle progression is regulated by mTOR through the p70S6K (ribosomal protein S6 kinase) and 4EBP1/eIF4E cascades [26,34]. Furthermore, active mTOR signaling has been associated with the development of HNSCCs [9]. The inhibition of S6 phosphorylation via mTORC1 inhibition can suppress but not wholly prevent 4EBP1/eIF4E cascades [35]. Indeed, this caused feedback activation of AKT signaling, suggesting that synergistic inhibition of PI3K/AKT and mTOR activation is a desirable therapeutic strategy to improve radiation therapy in HNSCCs [36]. 

It has been demonstrated that the S6K1 and 4EBP1/eIF4E signaling pathways are required for mTOR-mediated cell-cycle progression. Both S6K1 and 4EBP1/eIF4E have been linked to rapamycin-induced G1 cell-cycle progression [37]. By binding the 5′-cap structure of the mRNA and boosting the emergence of the translation initiation complex and ribosome binding, the eIF4E plays an important role in mRNA translation. Human and experimental cancers have been associated with high levels of eIF4E expression with links to angiogenesis and tumor growth. These increased levels in turn increase the expression of oncogenes c-myc and cyclin D1 (both components of cell-cycles) [38]. Previous findings strongly suggested that eIF4E suppression is also required for complete G1 cell cycle arrest. The cell-cycle phase distributions were analyzed to further assess the synergistic effects of these proteins in all tested OSCC cells after irradiation or integrated drug-irradiation treatment. Inhibiting the PI3K/AKT/mTOR signaling pathway causes cell cycle arrest in the G1 phase, according to our findings [36]. Furthermore, expression levels of the G1 phase regulators, cyclin D1 and CDK4, presented significant decreases. There is further evidence that mTOR inhibition is associated with G1 arrest. While this can be suppressed by 4EBP1 phosphorylation, mTOR inhibition cannot successfully suppress eIF4E in the majority of cells [35,36,39,40].

The current study conducted a tissue microarray (TMA) on 54 HNSCC patients who underwent definitive surgery and adjuvant radiotherapy. Both HNSCC-recurrent and HNSCC-nonrecurrent patients exhibited highly activated PI3K/AKT/mTOR signaling, as revealed by a TMA block with IHC staining. Low-expression phospho-eIF4E or high-expression eIF4E, phospho-4EBP1, phospho-S6K, and phospho-40S ribosomal protein S6 (p-S6R) was associated with reduced likelihood of survival for recurrent patients. Furthermore, expressions of eIF4E and p-4EBP1 were significantly correlated with tumor recurrence or recurrence-free survival. High expressions of eIF4E and p-4EBP1 were significantly correlated with low levels of recurrence-free survival. Finally, eIF4E and p-4EBP1 expression should be considered as predictive biomarkers for HNSCC patients [9,37]. 

## 3. Targeting of PI3K/AKT/mTOR Signaling for Radiosensitization

In human malignancies, it is common for the PI3K/AKT/mTOR signaling pathway to be dysregulated [41,42]. This pathway been connected to not only the onset and progression of malignancies but also the enhancement of cell survival, proliferation, and cellular metabolism. Currently, drugs that target PI3K or mTOR are undergoing clinical trials. There is already evidence that these drugs combined with radiation exhibit an improved anticancer effect. However, complex signaling events are induced by PI3K and mTOR. These events initiate a diverse range of functions associated with the regulation of cell survival and therapeutic resistance. It has been found that more PI3K/AKT/mTOR-related proteins are expressed in radioresistant OSCC cell lines than in parental cell lines [36]. This indicates that the PI3K/AKT/mTOR signaling pathway increases OSCC radioresistance. Radiosensitization can be induced by numerous small molecular drugs that target individual PI3K, AKT, or mTOR signaling proteins, or dual PI3K and mTOR blockade, [35,36,39,43,44]. The current paper focuses on the inhibitory effects of these drugs. Our results show that a combination of PI3K/AKT/mTOR inhibition and irradiation negatively affected irradiation-resistant cells, patient-derived OSCC cells, and other OSCC cell lines (Table 1).

### 3.1. mTOR Inhibitors

Everolimus (RAD001), an allosterically inhibitor of only mTORC1 but not mTORC2, is clinically used to treat various cancers, although the phase II clinical trial of everolimus in patient with previously treated recurrent or metastatic HNSCC is terminated [13,35,45,46,47]. Everolimus induces feedback activation of AKT signaling due to the inability to directly inhibit mTORC2 function, which can reduce their antitumor activity [35]. Dual mTORC1/mTORC2 inhibitor, AZD2014, conversely, increased the irradiation-induced inhibition of survival in both OSCC cell lines and patient-derived cells [36]. Previous research indicated that RAD001 and AZD2014 significantly increase radiation sensitivity by regulating cell cycle arrest, suggesting that they could be useful small-molecule drugs for patients with radioresistance [36]. AZD2014 dose-dependently enhanced the irradiation-induced cell death of OSCC cells [39]. AZD2014 and radiation can work synergistically to inhibit primary OSCC cell proliferation and induce cell death by inhibiting the AKT/mTOR pathway [64]. Moreover, treatment with AZD2014, combined with PI3K inhibitors BKM120 or BYL719, synergistically enhanced cell-growth inhibition by 4-Gy irradiation in radioresistant OSCC cells compared with each inhibitor combined with irradiation [44]. This suggests that combining mTOR and PI3K inhibiters with radiation inhibited the growth of radioresistant OSCC cells. The critical role played by the PI3K/mTOR signaling pathway in cellular mRNA translation and cell cycle progression demands further investigation into the effect of the triple-combination treatment. Our results confirm those of previous studies in conclusively proving that PI3K/mTOR signaling is a critical target for anticancer agents for radioresistant cells [21,35,39,43,44]. 

### 3.2. PI3K Inhibitors

Abnormal cells commonly survive, proliferate, and metabolize through the PI3K/AKT/mTOR signaling pathway [65]. Consequently, drugs can be used to target PI3K or mTOR to reduce tumor growth [66]. BKM120, an oral, highly specific pan-Class I PI3K inhibitor, has strong antiproliferative and synergistic characteristics in terms of radiosensitization in tumor cell lines [67,68], but it fails to improve cell killing after irradiation exposure [44]. Combining dual PI3K/AKT/mTOR inhibitors with irradiation, however, significantly increased irradiation-induced regression of OSCC cells. Indeed, the success of this combination treatment is on par with that of cisplatin with irradiation treatment. In addition, the PI3K inhibitor BKM120/BYL179 combined with radiation not only impacted the AKT/mTOR pathway, but also greatly reduced SCC4 and SCC25 cell survival. In sum, dual PI3K/mTOR inhibition is a promising avenue for future radiation research [36]. 

### 3.3. PI3K/mTOR Dual Inhibitors

PI3K/mTOR dual inhibitor, BEZ235, inhibited eIF4E and S6K phosphorylation and showed statistically significant antitumor activity and synergy with irradiation against OML1-R xenografts. During the study period, no significant weight loss or illness was noted, indicating that this therapy may have a promising safety profile. Furthermore, in vivo treatment with BEZ235 and irradiation is a safe and effective treatment that provided a greater therapeutic gain than radiation therapy alone [36].

Although eIF4E was de-repressed in BEZ235-treated OSCC cells, inhibiting the cyclin D1/CDK4 complex activity led to G1 cell cycle progression. These findings show that combining a dual PI3K/mTOR inhibitor with irradiation has a synergistic effect on repressing both S6K and 4EBP1/eIF4E signaling pathways and inducing G1 arrest by inhibiting cyclinD1 and CDK4 activities, resulting in increased sensitivity to irradiation [36]. These findings suggest that eIF4E is a logical therapeutic target for increasing tumor cell radiosensitivity and overcoming cancer radioresistance, implying that eIF4E-targeting strategies for oral cancer treatment may have clinical utility. 

γ-H2AX formation has previously been linked to the induction of double-strand breaks after exposure to irradiation or other DNA-damaging agents [69] and has also been linked to radiosensitization after mTOR inhibition [70]. However, there were no significant differences in the phosphorylation of γ-H2AX across BEZ235 treatments. Additionally, treatment with BEZ235 in combination with irradiation resulted in a slight increase in autophagy but had no effect on apoptosis in OSCC cells for any of the treatments [36]. It has been shown that autophagy exhibits a controversially cytoprotective or cytotoxic role in cancer radiation treatment [71], suggesting that dual inhibition of PI3K/mTOR may preferably promote radiosensitivity via an sautophagy-mediated pathway. Therefore, targeting of the PI3K/AKT/mTOR signaling pathway is a potential therapeutic strategy for irradiating OSCC, including patient-derived cells, OSCC radioresistant cell lines, and xenografts.

### 3.4. Inhibitors in Radiation-Resistant HNSCC Cells

We confirmed the radioresistant phenotype of the OML1-R cell line through the measurement of the plating efficiency of parental OML1 and radioresistant OML1-R subline cells. These were cultured following the exposure to high-dose fractionated irradiation (10 Gy). We assessed their survival using clonogenic survival assay. In comparison to parental cells, OML1-R cells showed substantially higher levels of clonal survival after irradiation [36]. 

To better understand the radioresistance of oral cancer cells and the role of the PI3K/AKT/mTOR signaling pathway in radiosensitization, inhibitors of the PI3K/AKT/mTOR pathway, such as BEZ235(Dactolisib), RAD001, and BKM120 (Buparlisib) [64], were used in radioresistant patient-derived OML-1R cells. The colony formation assay was used to assess the radiation sensitivity of PI3K/mTOR dual target inhibitor BEZ235 and mTORC1 inhibitor RAD001 in OML-1 and OML1-R cells exposed to 4Gy radiation [35]. The results revealed that when compared to the two cell lines exposed to 4Gy radiation alone or in combination with 4Gy radiation combined with RAD001 or BEZ235, the combined treatment of the two drugs and radiation could increase the radiation sensitivity of the two cell lines, with BEZ235 performing better. Therefore, BEZ235 is considered a better radiosensitizer than RAD001. BEZ235+4Gy radiation was also found to significantly inhibit the growth of OML-1 cell colonies when compared to 4Gy radiation alone (control group) and RAD001+4Gy radiation (RAD001 combined with 4Gy radiation). In OML-1R cells, RAD001+4Gy radiation inhibits cell growth only slightly more than 4Gy radiation alone; however, BEZ235+4Gy radiation inhibits OML-1R growth more effectively than RAD001+4Gy radiation. Furthermore, BEZ235+4Gy radiation can more effectively inhibit OML-1 growth. Because the radiation suppression effect is similar to that of OML-1 cells, BEZ235 could reverse radiation resistance [36]. Therefore, BEZ235 inhibits cell growth by reversing the radiation resistance effect of OML-1R cells, which may be regulated by PI3K molecules. G2/M checkpoint performance of OML-1R cells was significantly altered by BEZ235 combined with radiation treatment, primarily through phosphorylation of Chk2, to inhibit Cdc2 and cyclinB1 performance; however, neither RAD001 treatment nor OML-1 cells affected its alterations. Therefore, the BEZ235-regulated radiation-resistant strain may prompt Chk2 via PI3K molecules, affecting the G2/M cell cycle, which could be related to its mechanism of increasing radiation sensitivity and reversing radiation resistance [22,33]. 

Previously, we used a cell-cycle inhibitor (CDK4/6 inhibitor LEE011) to confirm the radiosensitization potential. LEE011 inhibited retinoblastoma protein phosphorylation, causing cell-cycle arrest in SCC4/SCC25 cells during the G1/M phase. LEE011 improved radiation effects in OML1 cells while overcoming radiation resistance in OML1R cells [43], suggesting that targeting of cell cycle serves as a radiosensitizer with the potential to enhance cytotoxicity.

## 4. Conclusions

**Figure 2:**
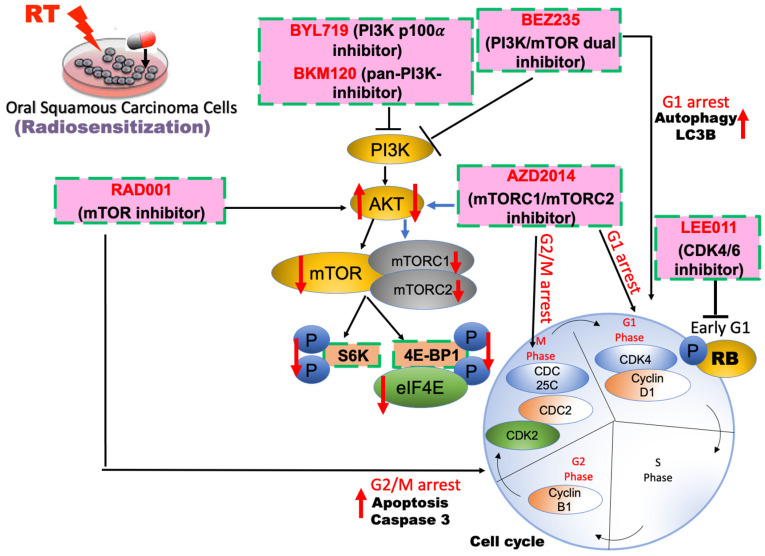
Schematic model of radiosensitizers via targeting PI3K/AKT/mTOR signaling in HNSCC.

In summary, the activation of the PI3K/AKT/mTOR signal pathway has been linked to radiotherapy resistance, and the effect of inhibitors of this pathway as a type of cancer radiosensitization is worth investigating (Figure 2). Inhibitors of PI3K/AKT/mTOR pathway not only increases the radiosensitivity of HNSCC cells but also reverses the radiation-sensitive effect of radiation-resistant cancer cells. In addition, cell cycle arrest increases cancer cell sensitivity to radiation. It is worth mentioning that the PI3K/mTOR dual-target inhibitor BEZ235 has a relatively good effect on radiation sensitization and reversal of radiation resistance, but it is also extremely toxic to normal cells and tissues, limiting its clinical application. Therefore, cancer treatment seeks an anticancer drug that is non-toxic to the human body and has no side effects. Radiation therapy is susceptible to resistance, and activation of the PI3K/AKT/mTOR pathway increases radiation resistance in HNSCC. It has become a hot topic, so inhibitors of this pathway cause radiation sensitization have been used for the development of new anticancer drugs. 

## Figures and Tables

**Table 1:** Inhibitors of PI3K/AKT/mTOR signaling pathway as radiosensitizers for the treatment of HNSCC.

Drug	Inhibitor	Mechanisms	Phase Ref.
Everolimus (RAD001)	mTORC1	Everolimus (RAD001) increased the radiosensitivity in SCC4 cells.	Phase I/II [13,35,45,46,47] (Afinitor^®^, Stein, Switzerland)
Vistusertib (AZD2014)	mTORC1/m TORC2 inhibitor	AZD2014 increased the irradiation-repressed cell viability of OSCC patient-derived cells and OSCS cell lines.	Phase II (Vistusertib^®^, Netherlands) [39,48,49,50]
Buparlisib (BKM120)	pan-PI3K inhibitor	BKM120 potently exhibited synergistic radiosensitization in OSCC cells.	Phase II [44,51,52,53]
Alpelisib (BYL719)	PI3Kp110𝛼 inhibitor	The combination of BYL719 with irradiation significantly enhanced irradiation-induced regression in OSCC cells.	Phase I [44,54,55,56,57]
Dactolisib (BEZ235)	PI3K/mTOR dual inhibitor	BEZ235 exhibited statistically antitumor activity with irradiation against OML1-R xenografts.	Phase I [33,58,59,60]
Ribociclib (LEE011)	CDK4/6 inhibitor	LEE011 enhanced the cytotoxic effects of radiation therapy in HNSCC cells.	Phase II [43,61,62,63]

## Data Availability

Not applicable.
